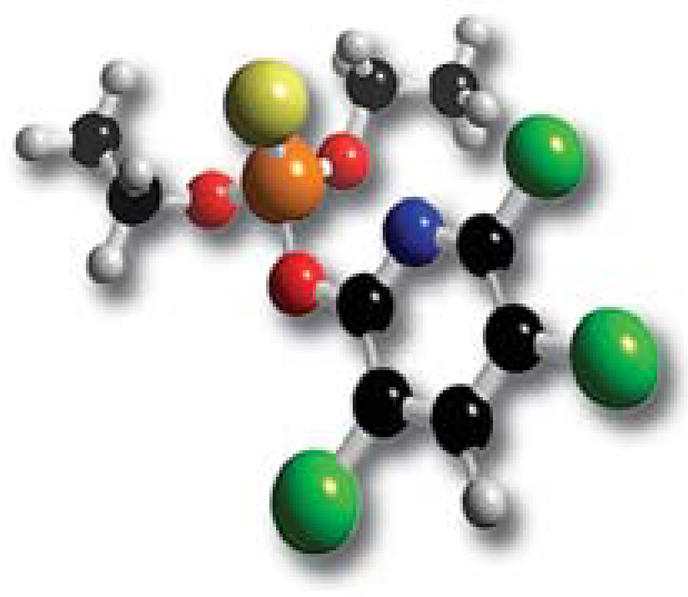# In Search of a Chlorpyrifos Antidote: Mechanisms Offer Clues

**Published:** 2007-09

**Authors:** Rebecca Renner

Treatments for acute organophosphate poisoning focus on stopping the buildup of the neurotransmitter acetylcholine at nerve endings. But organophosphates also interfere with neural cell development at much lower doses over a window of time that extends from fetal to neonatal development. This damage occurs through mechanisms that include direct interactions with acetylcholine receptors, interference with intracellular signaling cascades, and oxidative stress. Thus, any treatment aimed at stopping these sorts of effects would need to address those different mechanisms. Now researchers have advanced toward finding such an antidote **[*EHP* 115:1306–1313; Slotkin et al.]**.

The team tested four treatments on the neurodevelopmental effects of the organophosphate pesticide chlorpyrifos. Using pheochromocytoma (PC12) cells, a tumor cell line that displays the major phases of neurodevelopment targeted by the pesticide, the authors evaluated the different treatments in terms of DNA synthesis, cell number (as indicated by measured amounts of DNA), cell size (as indicated by the ratio of protein to DNA), and cell signaling mediated by adenylyl cyclase.

Because chlorpyrifos can cause adverse effects by allowing acetylcholine to build up at nerve endings, the scientists tested two receptor agonists, atropine (which blocks muscarinic receptors) and mecamylamine (which blocks nicotinic receptors). This treatment did not protect against the antimitotic action of chlorpyrifos: new cells failed to develop. However, once cells started to differentiate, the antagonists offered some protection against cell loss, although they could not prevent the deterioration of adenylyl cyclase signaling, which is essential for the development of neural networks.

The second treatment, nicotine, by itself had a small negative effect on the development of new cells, but it protected undifferentiated cells from the actions of chlorpyrifos and had mixed effects on cell numbers in differentiating cells. Nicotine both stimulates and blocks nicotinic receptors, and also possesses a mixture of pro- and antioxidant activity. The third treatment, the antioxidant vitamin E, also protected both undifferentiated and differentiating cells from many of the adverse effects of chlorpyrifos, but worsened the deterioration of adenylyl cyclase signaling. The fourth treatment, theophylline, a phosphodiesterase inhibitor that prevents the breakdown of cyclic adenosine monophosphate (the second messenger produced by adenylyl cyclase), was the only agent that restored cell signaling to normal or supranormal levels but did so at further cost to cell replication.

This new information indicates that it might be possible to design a cocktail of agents to counteract adverse neurodevelopmental effects of chlorpyrifos or other organophosphates, according to the authors. But they predict that finding an appropriate mix that avoids harmful effects inherent in the treatment agents, establishing doses, and determining whether the cocktail works for different chemicals will likely be a daunting task.

## Figures and Tables

**Figure:**